# Stent Retriever as Distal Filter for Endovascular Management of Acute Atherosclerosis-Related Carotid Occlusions: Preliminary Findings with a Novel Technique

**DOI:** 10.3390/jcm14041352

**Published:** 2025-02-18

**Authors:** Víctor Maestro, Eduardo Murias, Davinia Larrosa Campo, José Rodríguez Castro, Josep Puig, Juan Chaviano, Elena López-Cancio, Sergio Calleja, Pedro Vega

**Affiliations:** 1 Interventional Neuroradiology Department, Radiology, Hospital Universitario Central de Asturias, 33011 Asturias, Spain; victor_maceda@hotmail.com (V.M.); jorocas03@gmail.com (J.R.C.); peveval@yahoo.es (P.V.); 2 Department of Neurology, Hospital Universitario Central de Asturias (HUCA), Instituto de Investigación Sanitaria del Principado de Asturias (ISPA), 33011 Oviedo, Spain; davialc@gmail.com (D.L.C.); elenacancio@gmail.com (E.L.-C.); scallejap@gmail.com (S.C.); 3 Department of Radiology (CDI) and IDIBAPS, Hospital Clínic de Barcelona, 08036 Barcelona, Spain; jpuigmd@gmail.com; 4 Interventional Neuroradiology Department, Radiology, Hospital Universitario Central de Cáceres, 10004 Extremadura, Spain; juanchaviano.grajera@gmail.com; 5 Department of Funcional Biology, University of Oviedo, 33003 Oviedo, Spain

**Keywords:** stroke, stent retrievers, balloon guide catheter, carotid occlusion, atherosclerosis

## Abstract

**Objectives:** This study focuses on a novel endovascular technique for treating acute ischemic stroke caused by carotid artery occlusion (CAO) related to extracranial atherosclerosis, a condition typically associated with poor outcomes due to extensive brain infarction and high hemorrhagic risk. While stent retriever thrombectomy is effective for large-vessel occlusions, managing atherosclerosis-related CAO presents challenges. **Methods:** This retrospective analysis involved a cohort of 18 consecutive patients treated at our center using a new approach that employs a balloon guide catheter (BGC) to access the common carotid artery. Stent retrievers are used as distal filters, and angioplasty is performed through the stent pusher. The technique aims to reduce procedural time and prevent distal embolisms, which are common complications in traditional methods. **Results:** The results indicate that this approach improves intervention times, increases first-pass success rates, and decreases distal embolism occurrences compared to conventional techniques. It also effectively overcomes obstacles like the management of antiplatelet therapy and lengthy procedures. **Conclusions**: These preliminary findings demonstrate that using stent retrievers as filters with BGCs, without the need for aspiration catheters, may offer a safer and faster treatment option for atherosclerosis-related CAO. However, further research is required to confirm these findings and potentially establish this technique as the standard in clinical practice.

## 1. Introduction

Carotid artery occlusion (CAO) represents 6–15% of acute ischemic strokes and is linked to a poor clinical prognosis due to its association with large infarct volumes, an elevated risk of hemorrhagic transformation, and limited effectiveness of intravenous thrombolysis (IVT) [1,2]. Despite the advances in mechanical thrombectomy in recent years, the effective recanalization rate (eTICI 2B-3) in these types of situations, according to the most updated literature, is 76% [3].

Patients with carotid artery occlusion (CAO) presenting with a high clot burden, poor distal flow, and concurrent tandem occlusion of the middle cerebral artery tend to experience worse clinical outcomes following treatment with intravenous thrombolysis (IVT) [4,5]. Mechanical thrombectomy with stent retrievers or aspiration catheters has demonstrated efficacy in the treatment of large-vessel occlusions (LVO), as evidenced by several large-scale, randomized controlled trials. Consequently, the indications for this procedure are progressively expanding [6].

Stent retrievers seem to be less effective in treating intracranial atherosclerosis-related cerebral arterial occlusions (CAOs) compared to purely embolic emergent large vessel occlusions (ELVOs). This is due to the high incidence of reocclusion during endovascular treatment, even after successful initial recanalization with stent retriever thrombectomy in intracranial atherosclerosis-related large vessel occlusions (LVOs) [7]. The treatment of acute atherosclerosis-related CAO, especially when accompanied by intracranial thrombus, is associated with a worse prognosis, primarily due to the presence of small-sized distal emboli that damage brain tissue during carotid thrombus extraction or during angioplasty with stent implantation in the cervical segment. Additionally, these patients experience a longer time from puncture to recanalization compared to those with isolated intracranial occlusion. For patients with residual stenosis that impairs blood flow despite multiple attempts with stent retriever thrombectomy, alternative surgical strategies, including suction thrombectomy, chemical thrombolysis, balloon angioplasty, or permanent stent placement, may be considered [8,9]. However, the frequent application of these endovascular procedures can often result in infarctions due to complications like dissection, bleeding, and thrombosis.

The aim of this research was to present the results of a new method for treating acute atherosclerosis-related CAO that involves using a stent retriever or two stent retrievers in the intracranial circulation before manipulating the proximal area and then performing percutaneous angioplasty through the pusher of the devices to capture distal emboli and prevent damage from small-caliber thrombi.

## 2. Methods

### 2.1. Patient Enrollment

This study comprised a retrospective analysis of a cohort of 18 consecutive patients, with acute atherosclerosis-related CAO, including those with or without intracranial thrombi, who were treated between 30 June 2023 and 30 June 2024 at our center with a novel technique.

Patients underwent mechanical thrombectomy according to current guidelines and the hospital’s ischemic stroke protocol. The study was approved by the Ethical Committee of Investigation of the Principality of Asturias (Code CEImPA 2022.440).

We collected the following data: demographic characteristics and risk factors of the sample (age, sex, high blood pressure, diabetes, dyslipidemia, smokers, previous cerebrovascular accident or transient ischemic attack, coronary heart disease, and antithrombotic therapy), clinical and radiological characteristics (National Institute of Health Stroke Scale [NIHSS], Alberta Stroke Program Early CT Score [ASPECTS], occlusion type [tandem or isolated carotid occlusion], occlusion site [tICA, terminal internal carotid artery. MCA, middle cerebral artery. MCA 1, segment 1 of middle cerebral artery. MCA 2, segment 2 of middle cerebral artery], and intravenous (i.v.) fibrinolysis), and technical outcomes compared to the historical cohort from our center in this type of occlusions (time from symptom onset to arterial puncture, time from symptom onset to recanalization, procedure time, modified treatment in cerebral infarction [mTICI], true First-Pass Effect [tFPE], modified First-Pass Effect [tFPE], distal embolism in the same or new territory, and symptomatic intracranial hemorrhage [sICH])

### 2.2. Endovascular Treatment

The endovascular treatments were performed by a team of four interventional neuroradiologists, two of whom have more than 15 years of experience in this field.

The treatment involved the use of stent retrievers as distal filters, followed by a technique of common carotid catheterization using a balloon guide catheter (BGC, FG2; Stryker Neurovascular, Fremont, CA, USA) and microcatheter (Phenom 21, Irvine, CA 92618, USA) crossing of the carotid obstruction. An exchange technique was utilized for microcatheter retrieval, and transluminal percutaneous angioplasty was performed in the proximal carotid with the administration of 900 mg of lysine acetylsalicylate. The procedure then advanced to the distal internal carotid artery (ICA), where mechanical thrombectomy of the intracranial arteries was performed using stent retrievers, concluding with carotid stent implantation in the proximal atheromatous plaque.

**Figure 1:**
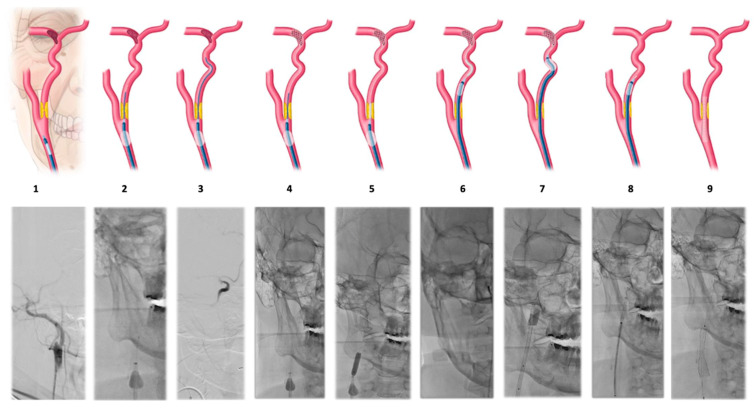
Step-by-step technique illustration.

Eighteen consecutive patients were treated by simplifying the technique described initially by Behme D et al. [10] without utilizing aspiration catheters. The procedure involved the catheterization of the common carotid artery (CCA) with a BGC to confirm carotid obstruction at the origin of the ICA. The balloon was then inflated in the CCA and a microcatheter was used to cross the atheromatous plaque at the ICA origin. A gentle injection confirmed the presence of thrombi in the ICA and determined the presence or absence of thrombi in intracranial arteries. Once the distal thrombus was located, the microcatheter was positioned distal to the last thrombus and a stent retriever was deployed, spanning the middle cerebral artery (MCA) and distal ICA. The stent retriever served as a filter to prevent distal embolisms during proximal manipulation. The next step involved withdrawing the microcatheter and using the stent retriever pusher as a guide to advance the angioplasty balloon to dilate the proximal atheromatous plaque before administering another angioplasty balloon. At this point, the BGC balloon was deflated, and continuous aspiration was performed through the same catheter to remove intracranial thrombi and the distal ICA. Then, the BCG was advanced to the subpetrosal ICA, and a pass was made with the previously opened stent retriever. Finally, a carotid stent was implanted to maintain plaque opening (Table 1 and Figure 1).

### 2.3. Objectives

The objectives are to present the results of a new technique for treating acute atherosclerosis-related CAO (using a stent retriever or two stent retrievers in the intracranial circulation before manipulating the proximal area and then performing percutaneous angioplasty through the pusher of the devices to capture distal emboli before performing the pass with stent retriever) and compare it with a historical cohort from our institution (using an antegrade approach, which involves, initially, the direct aspiration of the carotid occlusion with a BGC and angioplasty to facilitate access to the intracranial thrombus, which was retrieved using a single stent retriever).

### 2.4. Statistical Analysis

Continuous variables are described as means (±SD) if the distribution was normal or otherwise as medians (IQR). Categorical variables are described as proportions (%). Student’s *t*-test is used for the continuous variables and a chi-square test is used for the contingency tables of the categorical variables, which, if the value of any of the boxes is less than or equal to 5, is replaced by a Fisher’s exact test. *p* < 0.05 has been considered statistically significant.

## 3. Results

The cohort has a mean age of 73 years, with 55.5% being males. In total, 72.2% of the patients had high blood pressure (HBP), 50% of the cohort had dyslipidemia, 33.3% were under antithrombotic therapy (anticoagulation or antiplatelet treatment), and 27.7% of them had diabetes. Furthermore, 22.2% of the patients were smokers, 16.6% had previous coronary heart disease and only 11.11% of the patients had suffered a previous cerebrovascular accident (CVA) or transient ischemic attack (TIA). These variables are presented in Table 2.

The mean NIHSS was 14 and APECTS was 7. A total of 16 patients (88.8%) in the cohort had a tandem occlusion, with the majority, 13 (72.2%), having associated thrombus in the middle cerebral artery, and only 2 (11.1%) had an isolated carotid artery occlusion (CAO). Among the intracranial occlusions, the majority, 10 (55.5%), were in the M1 segment of the MCA, 3 (16.6%) in the M2 segment of the MCA, and another 3 (16.6%) in the terminal segment of the internal carotid artery (tICA). Only one patient (5.5%) received intravenous (i.v.) fibrinolysis. These results are shown in Table 3.

Table 4 represents the comparative analysis of technical outcomes and procedure times between the study sample corresponding to group 1 and a historical cohort from our institution in this type of arterial occlusion [11], represented in group 2.

Time from symptom onset to arterial puncture: group 1 had a mean time of 267 min (±133) from symptom onset to arterial puncture, while group 2 showed a slightly shorter mean time of 207.12 min (±88.1). These findings were not statistically significant (*p* = 0.087).

Time from symptom onset to recanalization: group 1 had a mean time of 297 min (±140), whereas group 2 had a mean time of 263.78 min (±89.3), with no statistically significant difference (*p* = 0.278).

Procedure time: group 1 had a mean procedure time of 29.8 min (±17.6), whereas group 2 had a mean time of 56.36 min (±19.3). These findings were statistically significant (*p* = 0.000).

Modified Thrombolysis in Cerebral Infarction (mTICI): mTICI 3—group 1 had 14 (77.7%) versus group 2, which had 45 (45.4%), with a statistically significant difference (*p*-value 0.012). mTICI 2B-3—group 1 had 15 (83.3%) versus group 2, which had 87 (87.8%), with a statistically significant difference (*p*-value 0.03). mTICI 2C-3—group 1 had 16 (88.8%) versus 76 (76.7%), with no statistically significant difference (*p*-value 0.080).

Further, the true First-Pass Effect (tFPE) in group 1 was 14 (77.7%) versus 48 (48.5%) with a statistically significant difference (*p*-value 0.012), and the modified First-Pass Effect (mFPE) was 15 (83.3%) versus 43 (43.4%), with a statistically significant difference (*p*-value 0.010).

Related to distal embolism in the same territory, group 1 had 3 (16.6%) while group 2 had 16 (16%) without any statistically significant difference (*p*-value 0.957). Distal embolism in the new territory in group 1 was 1 (5%) while in group 2 it was 8 (8%), with no statistically significant difference (*p*-value 0.711).

Group 1 had no sICH, 0 (0%) while group 2 there were 12 (12.1%), with a statistically significant difference (*p*-value 0.000).

## 4. Discussion

In our review of the outcomes for patients who experienced an ischemic stroke due to an atheromatous plaque at the beginning of the internal carotid artery (ICA), we noticed some difficulties when compared to the results seen with the middle cerebral artery (MCA). The first issue was related to the use of antiplatelet agents due to our group’s preference for carotid stent use. This problem was resolved by implementing mono-aggregation with stent implantation and adjusting antiplatelet therapy using dual-energy CT scans [12]. Another challenge encountered was that the procedure time was longer than that of other locations and etiologies and the methodology was more complicated than that of isolated M1 occlusions.

Furthermore, many patients exhibited TICI 2B-3 at the end of the procedure, but there was no clinical correlation due to the presence of distal emboli in the brain parenchyma. Several techniques have been proposed to address these two problems but many of them involve the use of intermediate aspiration catheters [13,14,15,16,17].

This technique employs the stent retriever pusher as a guide for angioplasty balloons, eliminating the need for one or two device exchanges. This not only streamlines the procedure but also improves intervention times and increases first-pass success rates. Additionally, the use of a distal stent retriever, aided by the proximal BGC, prevents thrombus migration during the manipulation of the ICA plaque, thereby reducing distal embolisms in at least new territories.

From a technical standpoint, the utilization of proximal BGC appears to be essential in controlling distal thrombus migration to the greatest extent possible through inflation, deflation, and aspiration. The chosen stent retriever should be as lengthy as feasible, with a pusher that is compatible with angioplasty balloons. In our study, we employed the Catch Maxi 6 × 30 mm (Balt Group, Montmorency, France) and Trevo TroVue 6 × 25 mm (Stryker Neurovascular, Fremont, CA, USA) stent retrievers, with a total extractor length of 40 cm, and their pushers were compatible with a Sterling balloon dilatation catheter 5 × 20 mm (Boston Scientific, Marlborough, MA, USA).

Behme D et al. [10] and Sultan-Qurraie A. et al. [18] described a similar technique for acute atherosclerosis-related CAO, but with our technique, we do not use aspiration catheters because we do not use them routinely for acute ischemic stroke since our strategy for ischemic stroke is a BGC and stent retriever, and unlike Sultan-Qurraie A. et al. [18], we always use a carotid stent at the end of the procedure through the BGC to maintain the plaque opening.

Although our study was preliminary in nature, the use of this method, without an aspiration catheter, has led to a significant reduction in intervention times, with approximately a 27 min reduction in time compared to the usual method used in our center. At this point, it is important to clarify that there is no significant difference in “time from symptom onset to arterial puncture” and “time from symptom onset to recanalization”, which highlights the speed of the technique with an improved first-pass effect (tFPE and mFPE), a greater degree of recanalization mTICI 3 and mTICI 2C-3, and a decrease, at least, in distal embolisms in the new territory (Table 4). Nevertheless, given the small number of patients included in the cohort and the statistical power underlying our analysis, these results, although promising, should be interpreted with caution.

Compared to similar literature [13,14,15,16,17,18], our recanalization rates are higher and are achieved in less time, which has compelled us to publish our results even though similar techniques have been described.

These results, in this type of patient with such a poor prognosis, have led us to consider that the use of a stent retriever as a distal filter should be used as a first-line approach due to its shorter procedure time, higher recanalization rate, and safety. Nonetheless, it is important to approach the lack of sICH in this study with caution, as the patient sample size was quite small.

Therefore, we propose this method, which uses a stent retriever as a filter during the treatment of acute atherosclerosis-related CAO in patients with ischemic stroke, as a potentially effective technique and deserving of consideration as the treatment of choice in these situations because of the reduction intervention times, the reduction in distal embolisms in at least new territories, and first-pass success rates.

The main limitation of our study is the relatively small sample, but this study serves as a preliminary exploration of a technique aimed at simplifying endovascular procedures.

To truly validate the findings and enhance clinical outcomes, further research with a larger group of patients and studies with greater scientific evidence will be necessary.

## Figures and Tables

**Table 1:** Step-by-step technique description.

Step	Description
1	Common carotid artery (CCA) catheterization with a balloon-guiding catheter (BGC).
2	Inflation of the balloon in the CCA and cross the atheromatous plaque at the initiation of the internal carotid artery (ICA) with a microcatheter and the micro guidewire.
3	Make a run through the microcatheter to confirm the presence of thrombi in the ICA and assess thrombi in intracranial arteries.
4	Deployment of a stent retriever at MCA and distal ICA.
5	Remove the microcatheter and advancement of the angioplasty balloon using the stent retriever pusher and dilation of the proximal atheromatous plaque.
6	Deflate the BGC balloon; continuous aspiration through the same catheter with a 60cc syringe. Advance the BGC towards the subpetrosal ICA with the support of the angioplasty balloon.
7	BCG is inflated again to make a thrombectomy pass with the stent retriever used as distal filter.
8	Distal to the atheromatous plaque through the BGC and with a 0.014 guidewire, (Traxcess, MicroVention Inc., Tustin, CA, USA) raise the carotid stent.
9	The carotid stent is implanted to maintain plaque opening.

**Table 2:** Demographic characteristics and risk factors of the sample.

*n*	18
Age, years	73 ± 8.7
Males	10 (55.5)
HBP *	13 (72.2)
Diabetes	5 (27.7)
Dyslipidemia	9 (50.0)
Smokers	4 (22.2)
Previous CVA ^†^/TIA ^‡^	2 (11.1)
Coronary heart disease	3 (16.6)
Antithrombotic therapy	6 (33.3)

Continuous variables are described as means (±SD) if the distribution was normal or otherwise as medians (IQR). Categorical variables are described as proportions (%). * HBP, high blood pressure. ^†^ CVA, cerebrovascular accident. ^‡^ TIA, transient ischemic attack.

**Table 3:** Clinical and radiological characteristics of the sample.

NIHSS *	14 (12–19)
ASPECTS ^†^	7 (7–8)
Tandem occlusion	16 (88.8)
Isolated carotid artery occlusion	2 (11.1)
tICA ^‡^	3 (16.6)
MCA ^§^	13 (72.2)
MCA 1 ^ǁ^	10 (55.5)
MCA 2 ^¶^	3 (16.6)
i.v. fibrinolysis	1 (5.5)

Continuous variables are described as means (±SD) if the distribution was normal or otherwise as medians (IQR). Categorical variables are described as proportions (%). * NIHSS, National Institute of Health Stroke Scale. ^†^ ASPECTS, Alberta Stroke Program Early CT Score. ^‡^ tICA, terminal internal carotid artery. ^§^ MCA, middle cerebral artery. ^ǁ^ MCA 1, segment 1 of middle cerebral artery. ^¶^ MCA 2, segment 2 of middle cerebral artery.

**Table 4:** Technical outcomes of the sample reported in group 1 compared to the historical cohort reported in group 2.

Characteristic	Group 1 (*n* = 18)	Group 2 (*n* = 99)	*p*-Value
Time from symptom onset to arterial puncture, min	267 ± 133	207.12 ± 88.1	0.087
Time from symptom onset to recanalization, min	297 ± 140	263.78 ± 89.3	0.278
Procedure time, min	29.8 ± 17.6	56.36 ± 19.3	0.000
mTICI * 3	14 (77.7)	45 (45.4)	0.012
mTICI 2B-3	15 (83.3)	87 (87.8)	0.003
mTICI 2C-3	16 (88.8)	76 (76.7)	0.080
tFPE ^†^	14 (77.7)	48 (48.5)	0.012
mFPE ^‡^	15 (83.3)	43 (43.4)	0.010
Distal embolism in the same territory	3 (16.6)	16 (16)	0.957
Distal embolism in the new territory	1 (5)	8 (8)	0.711
sICH ^§^	0 (0)	12 (12.1)	0.000

Continuous variables are described as means (±SD) if the distribution was normal or otherwise as medians (IQR). Categorical variables are described as proportions (%). Student’s *t*-test is used for the continuous variables and the chi-square test is used for the contingency tables of the categorical variables, which, if the value of any of the boxes is less than or equal to 5, is replaced by a Fisher’s exact test. *p* < 0.05 is considered statistically significant. * mTICI, modified treatment in cerebral infarction. ^†^ tFPE, true First-Pass Effect. ^‡^ mFPE, modified First-Pass Effect. ^§^ sICH, symptomatic intracranial hemorrhage.

## Data Availability

No new data were created. All data are shown in the article.
